# PSMC-FAC: Automated Optimization of False-Negative Rate Corrections for Low-Coverage PSMC-Based Demographic Inference

**DOI:** 10.3390/biology15080631

**Published:** 2026-04-16

**Authors:** Francisco Iglesias-Santos, Alba Nieto, Sònia Casillas, Antonio Barbadilla, Carlos Sarabia

**Affiliations:** 1 Institut de Biotecnologia i Biomedicina (IBB), Universitat Autònoma de Barcelona (UAB), 08193 Bellaterra, Spain; francisco.iglesias.santos@univie.ac.at (F.I.-S.); sonia.casillas@uab.cat (S.C.); antonio.barbadilla@uab.cat (A.B.); 2 Department of Mathematics, University of Vienna, 1090 Vienna, Austria; 3 Department of Evolutionary Biology, University of Vienna, 1010 Vienna, Austria; 4 RIKEN Center for Interdisciplinary Theorethical and Mathematical Sciences (iTHEMS), RIKEN, Wako 351-0198, Japan; 5 Departament de Genètica i de Microbiologia, Universitat Autònoma de Barcelona (UAB), 08193 Bellaterra, Spain; 6 Institut de Biologia Evolutiva, Universitat Pompeu Fabra (IBE-UPF), 08003 Barcelona, Spain

**Keywords:** demographic inference, Sequential Markovian Coalescent, PSMC, Hausdorff distance, Fréchet distance, low-coverage genome

## Abstract

Inferring demographic history from whole-genome data is a central objective in evolutionary and conservation genomics. However, the Pairwise Sequentially Markovian Coalescent (PSMC) framework, one of the most widely used demographic inference methods for whole-genome sequence data, is highly sensitive to sequencing coverage, with low coverage producing systematic underestimation of heterozygosity, which biases effective population size trajectories. Here, we present PSMC-FAC, an automated method designed to optimize false-negative rate correction in low-coverage genomes by minimizing geometric distances between FNR-corrected low-coverage trajectories and their corresponding high-coverage references. Whole-genome datasets from humans, gray wolves, and cattle were downsampled across multiple coverage levels and processed through standard demographic inference pipelines. Corrected trajectories, projected onto a common temporal grid, were compared using Hausdorff and discrete Fréchet distance metrics and optimal correction factors were modeled as a function of sequencing depth using second-degree polynomial regression. Across species and demographic contexts, PSMC-FAC substantially improved concordance between low- and high-coverage trajectories and revealed highly predictable coverage-dependent correction patterns. Overall, PSMC-FAC provides a reproducible and mathematically grounded alternative to subjective correction approaches, enabling reliable demographic inference from moderate-coverage genomes and facilitating broader population-scale genomic analyses.

## Simple Summary

Understanding how populations across the tree of life changed over time helps scientists explain evolution, protect biodiversity, and predict how species may respond to environmental change. Modern DNA sequencing makes it possible to study population history, but collecting very detailed genetic data is expensive and often not practical. Researchers therefore frequently use lower-quality genetic data, which can lead to inaccurate results because some genetic differences are missed. In this study, we present a new automated method that quantifies and compensates for missed heterozygous sites, adjusting the results accordingly. The approach uses geometric distance minimization to identify the optimal correction, avoiding subjective choices by researchers. Validated across genomic data from humans, grey wolves, and cattle—species with distinct demographic histories—the method yields robust, reproducible corrections and substantially improves agreement between low- and high-coverage demographic trajectories. PSMC-FAC reduces sequencing cost requirements while extending the reach of demographic inference across evolutionary biology, ecology, and conservation genomics.

## 1. Introduction

Reconstructing species’ demographic histories is a main goal in evolutionary biology and conservation genetics, providing insight into how populations responded to past climatic fluctuations, geological events, and anthropogenic pressures, and helping predict future responses. Classical genetic markers, such as mitochondrial DNA and microsatellites, yield valuable information about recent population dynamics, but their limited temporal resolution restricts demographic inference largely to the most recent thousands of years [1,2,3,4]. In contrast, whole-genome sequencing (WGS) has driven the emergence of population genomic approaches capable of inferring demographic change across hundreds of thousands of years. Among these, the Pairwise Sequentially Markovian Coalescent (PSMC) method represents a widely adopted advance, allowing the reconstruction of effective population size (Ne) trajectories from a genome across deep evolutionary timescales [5].

PSMC infers changes in Ne through time by modeling genome-wide patterns of heterozygosity, which reflect variation in the time to the most recent common ancestor (TMRCA) between homologous genomic regions. Because demographic contractions, expansions, and periods of stability leave distinct signatures in these patterns, PSMC provides a powerful framework for investigating long-term population history under a coalescent-based model. The method, although dependent on generation time and mutation rate, is particularly informative for timescales spanning approximately 10,000 to 1–3 million years ago and has been widely applied across diverse taxa, including humans and both wild and domesticated species [5,6,7,8,9].

The theoretical foundation of PSMC lies in coalescent theory, which provides a retrospective description of genealogical relationships by tracing sampled lineages backward in time until they merge at common ancestors [10,11]. In recombining genomes, genealogies vary along the sequence, forming a complex structure known as the ancestral recombination graph (ARG). Although the ARG provides a complete representation of ancestry, its high dimensionality makes it computationally intractable for most practical applications [12]. To overcome this limitation, approximations based on Hidden Markov Models have been developed, leading to the Sequentially Markov Coalescent (SMC), which assumes that genealogical changes along the genome follow a Markov process [12,13]. Building on this framework, PSMC models coalescent time at each genomic position as a hidden state and the observed homozygous or heterozygous genotype as the emission, enabling inference of Ne trajectories from a single genome that, under ideal assumptions, can approximate the history of an entire population [14,15].

Despite its broad utility, largely due to its non-parametric nature, PSMC is sensitive to data quality, demographic complexity, and analytical choices. PSMC further assumes neutrally evolving regions within a panmictic population. However, background selection or the inclusion of overlooked constrained genomic elements can bias estimates of coalescent rates and effective population size trajectories [16,17]. Population structure can be another source of bias. Past coalescent rate depends on migration and deme configuration in addition to changes in Ne [18,19], making interpretation of inference non-trivial. Beyond this change in expected curve due to population structure, PSMC may generate spurious, abrupt peaks under structured scenarios that do not reflect the underlying coalescent dynamics [20]. Similar instabilities can also arise from misspecification of the PSMC time-interval discretization (“time vector”) [21]. Although extensions such as MSMC and SMC++ improve resolution by incorporating multiple genomes [22,23], they retain the panmixia assumption, along with biases associated with this assumption [20]; only more recent developments explicitly accommodate structured demographic scenarios [24]. However, the incorporation of structured scenarios in SMC-based inferences remains a field in development.

Inferring population-wide demographic history in natural populations is especially difficult when population structure is ubiquitous [25,26]. Typically, multiple genomes are required to characterize underlying demographic processes and disentangle biological signals from methodological artefacts [20]. Consequently, empirical studies frequently adopt low- to medium-coverage sequencing strategies and prioritize sampling large numbers of individuals to better capture population-level variation. When considering genome data quality, sequencing errors, false-negative heterozygous calls, and insufficient coverage can bias heterozygosity estimates and distort inferred demographic histories [6,9]. A particularly important limitation of the PSMC method arises when using low-coverage genomes. Reduced coverage decreases detection of heterozygous sites, leading to systematic underestimation of individual heterozygosity and consequently biased inference of Ne and coalescent times. PSMC trajectories are consequently displaced downward and shifted toward more recent timescales while sharp demographic changes appear attenuated and temporally shifted [4]. One strategy to mitigate this bias during plotting of the PSMC curve is to estimate the false-negative rate (FNR), a statistical correction for lost heterozygosity through rescaling of inferred coordinates from the first estimation of genetic diversity. Traditionally, FNR has been estimated by downsampling a high-coverage genome to lower coverage, plotting both together, and visually adjusting FNR values of the low-coverage curve until it partially overlaps with the high-coverage reference curve [7,8,9,27]. Although this approach allows incorporation of more genomes and improves PSMC-based inferences, visual corrections are subjective, time-consuming, difficult to reproduce and non-transferable across laboratories. To our knowledge, no previously published automated framework exists for optimizing PSMC FNR corrections. Because generating large numbers of high-coverage genomes remains cost-prohibitive for many laboratories, population genomics will increasingly rely on medium- and low-coverage datasets. Consequently, there is a growing need for fast, objective, and mathematically grounded approaches to estimate and evaluate FNR corrections and enable robust comparisons of demographic trajectories across samples.

Here, we present PSMC FNR-Automatized Correction (PSMC-FAC), a novel method that introduces a statistical framework for optimizing FNR correction of low-coverage PSMC trajectories by minimizing geometrical distances between candidate FNR-corrected low-coverage curves and their corresponding high-coverage reference curves. We assessed this approach using downsampled high-coverage publicly available WGS data from multiple populations of humans (Homo sapiens), a wild species (European gray wolves, Canis lupus), and a domesticated species (cattle, Bos taurus), representing diverse demographic histories and varying levels of genome-wide heterozygosity. We show that PSMC-FAC provides reproducible and robust FNR estimates through an automated pipeline applicable to any WGS data in BAM format, independent of genome-wide heterozygosity and demographic history. By enabling accurate demographic inference from low-coverage genomes, PSMC-FAC substantially lowers sequencing cost requirements and broadens access to population genomic analyses, thereby facilitating large-scale and comparative studies of demographic history across a wide range of taxa.

## 2. Materials and Methods

### 2.1. Dataset Preparation

We downloaded previously published WGS data for multiple populations of interest from publicly available repositories, including humans, cattle, and wolves. Wolf genomes were obtained in FASTQ format [28] and aligned to the CanFam3.1 reference genome [29] using bwa-mem v0.7.17 [30]. Reads were filtered to remove spurious alignments and PCR duplicates and were sorted with several samtools functions (samtools view -f2 -F260, samtools dedup and samtools sort). Human WGS data were downloaded in CRAM format [31] from the 1000 Genomes Project [32] and included nine individuals from three different geographical origins: three Han Chinese (CHB), three Yoruba from Ibadan, Nigeria (YRI), and three Tuscans from Italy (TSI). Files in CRAM format were decompressed and transformed to BAM format using samtools [33]. Cattle genomes were obtained in BAM format from the public Agricultural Research Service of the United States Department of Agriculture [34,35]. Genome-wide sequencing depth for all samples was calculated using the samtools depth function [36]. Samples with mean coverage >18× were retained to enable downsampling. Metadata for all downloaded samples are provided in Table A1.

Downsampling was performed on all high-coverage BAM files to multiple target depths (5×–15×) using the samtools view -s function, representing a range of low-to-medium depths normally found in library preparations from samples with suboptimal DNA quality found in the field [27]. Genome-wide depth of coverage for each downsampled BAM file was verified using the samtools depth function (Table A1). Following the PSMC manual specifications [5], downsampled BAM files were converted to variant call format (VCF) using bcftools mpileup and call functions [36]. To run this step, the CanFam3.1 reference genome [29] was used for wolves, the GRCh38 (hg38) reference genome [37] for humans, and the bosTau9 reference genome [38] for cattle. Variant Call Format (VCF) files [39] were then converted to FASTQ format using the bcftools view function and the vcfutils.pl vcf2fq utility [5], filtering out variants with coverage lower than 5 (-d 5) and higher than twice the average depth of coverage (-D), retaining most heterozygous sites while filtering out gene duplicates. Finally, FASTQ files were converted to PSMC input format (PSMCFA) using fq2psmcfa [5] with a minimum phred-scaled base quality threshold of 20.

**Figure 1:**
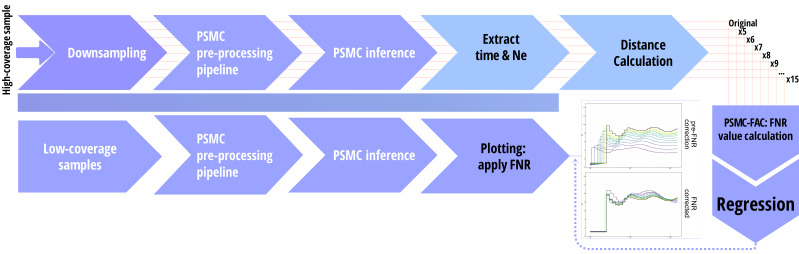
**Schematic overview of the PSMC-FAC pipeline.** A high-coverage genome (e.g., 20×) in BAM format from a given population is downsampled to multiple lower coverages (5×–15×), and each dataset is processed through the standard PSMC workflow. PSMC-FAC then estimates the optimal False Negative Rate (FNR) for each coverage level by minimizing the Hausdorff and discrete Fréchet distances between the original and downsampled trajectories. The resulting optimal FNR values are plotted against sequencing coverage, and a polynomial regression is fitted. This regression can subsequently be used to infer appropriate FNR corrections for additional genomes from the same population sequenced at varying depths.

We obtained a PSMC inference from each individual sample and its downsamples and followed the literature to define time interval patterns. For wolves, we applied a customized time interval pattern of 64 atomic intervals arranged as six intervals of size one followed by 58 intervals of size one (1*6+58*1), as described in Freedman et al. [40]. For humans and cattle, we used the default PSMC time interval pattern (4+25*2+4+6), following Li and Durbin (2011) [5] for humans and Mei et al. (2018) [41] and Liu et al. (2020) [42] for cattle (see Appendix A for specific commands and the pipeline in Figure 1).

For each PSMC trajectory inferred from a downsampled genome, we applied False Negative Rate (FNR) correction across values ranging from 0 (no correction) to 0.99 in increments of 0.01, generating 100 corrected PSMC trajectories per downsample. Each corrected trajectory was then compared to the PSMC curve inferred from the original (non-downsampled) sequence. Because PSMC trajectories are defined over discretized time intervals that differ between inferences, direct pointwise comparison is not straightforward. To standardize comparisons, we defined a custom vector of 60 logarithmically spaced time points (in years) and projected all trajectories onto these shared temporal coordinates.

For cattle and wolves, the custom vector spanned 10 kya to 1.5 Mya. For humans, we restricted the analysis to the range of 50 kya to 1.5 Mya to avoid recent-time instabilities in PSMC. Early analyses attributed these peaks to Out-of-Africa demographic history [5], whereas more recent work has shown that they can arise from population structure [20] and from sensitivity to time-interval discretization [21]. Restricting the lower bound from 10 to 50 kya was done to mitigate the influence of these artifacts.

### 2.2. A Mathematical Approach to Compute FNR for Low-Coverage Samples

To quantify differences between PSMC trajectories, we employed two complementary distance-based metrics: (i) Hausdorff distance [43] and (ii) the discrete Fréchet distance [44] (1). Each PSMC trajectory is represented as an ordered set of points in the plane, where the x-coordinate corresponds to log-scaled time and the y-coordinate corresponds to inferred effective population size (Ne). Let

(1) $$P=\left\{p_{1},p_{2},\ldots ,p_{m}\right\},\;Q=\left\{q_{1},q_{2},\ldots ,q_{m}\right\}$$

denote two PSMC curves, where P represents the reference trajectory obtained from a high-coverage genome (for example, 20×) and Q represents a trajectory derived from a downsampled genome after FNR correction. Before distance computation, trajectories are projected onto a shared temporal grid so that corresponding demographic histories can be compared consistently while preserving the piecewise-constant nature of PSMC inference.

### 2.3. Hausdorff Distance

The undirected Hausdorff distance (2) measures the maximal local discrepancy between two curves. It is defined as

(2) $$H\left(P,Q\right)=\max\left\{\max_{p_{i}\in P}\min_{q_{j}\in Q}∥p_{i}-q_{j}∥,\;\max_{q_{j}\in Q}\min_{p_{i}\in P}∥q_{j}-p_{i}∥\right\}$$

where ∥·∥ denotes the Euclidean norm [43].

For each point in one curve, the minimum distance to the other curve is computed. The Hausdorff distance corresponds to the largest of these minimal distances and therefore captures the worst-case disagreement between trajectories. This metric is sensitive to localized deviations, such as abrupt expansions or bottlenecks, and is useful for identifying regions where curves diverge strongly.

#### 2.3.1. Discrete Fréchet Distance

While the Hausdorff distance ignores the sequential ordering of points, demographic trajectories represent ordered processes through time. To account for temporal progression, PSMC-FAC additionally uses the discrete Fréchet distance (3), which preserves ordering along both curves and measures similarity in overall shape. The discrete Fréchet distance between two polygonal curves is defined as

(3) $$\delta_{F}\left(P,Q\right)=\min_{\sigma ,\tau }\max_{k=1,\ldots ,m}∥P_{\sigma (k)}-Q_{\tau (k)}∥$$

where σ and τ are non-decreasing index sequences that traverse the points of P and Q, respectively, from start to end, preserving ordering [44].

Intuitively, the Fréchet distance measures the minimum leash length required for two entities to traverse both curves without backtracking. Because temporal ordering is maintained, this metric captures global similarity in demographic trajectory shape rather than isolated local differences.

#### 2.3.2. A Combination of Both Methods

By using both metrics, we can detect localized deviations and global differences in curve geometry, providing a more comprehensive and robust comparison than either metric alone (Appendix A). A custom distance-calculation script computed both the discrete Fréchet distance and the Hausdorff distance between the original path and each FNR-corrected path.

After evaluating the accuracy of both the Hausdorff and Fréchet distances for our samples, we defined the optimal FNR value for each downsampled genome as the FNR corresponding to the minimum discrete Fréchet distance relative to the original (high-coverage) trajectory. The Fréchet metric was selected as the primary optimization criterion because it preserves point ordering and thus reflects similarity in overall demographic trajectory shape and temporal progression. Hausdorff distances were retained to evaluate localized maximal deviations.

The set of optimal FNR values obtained across coverage levels was subsequently modeled using a polynomial regression of degree to generate continuous correction curves. Polynomial models of degree 1–3 were evaluated, and a second-degree polynomial was selected as it provided the best fit (highest R2) while maintaining model parsimony. These fitted polynomial functions describe the relationship between sequencing depth and the FNR value that minimizes trajectory divergence, thereby enabling depth-dependent calibration of Ne estimates.

### 2.4. FNR and Heterozygosity as a Function of Coverage

We evaluated whether the optimal FNR corrections depend not only on genome-wide heterozygosity and sequencing coverage, but also on the demographic history of the population under study. To assess this, we systematically examined the relationship between sequencing depth and the most optimal FNR factor inferred for each individual. To model the relationship between sequencing depth and optimal FNR, we fitted a second-degree polynomial regression in which coverage was treated as the independent variable, and the most optimal FNR value as the dependent variable. This polynomial model was chosen to capture non-linear scaling of FNR with sequencing depth. The regression was fitted independently for each individual.

Because optimal FNR values were determined using two alternative distance metrics, we repeated the polynomial fitting procedure separately for FNR estimates obtained through minimization of the Hausdorff distance [43] and for those obtained through minimization of the Fréchet distance [44], allowing comparison between both metrics. Following Nadachowska-Brzyska et al. [9], we assumed that the optimal FNR is equal to zero for samples with mean coverage of 15×. Accordingly, for regression purposes, high-coverage genomes exceeding this threshold were treated as having an optimal FNR=0. This anchors the fitted relationship at high coverage and reflects the expectation that false negative errors become negligible as sequencing depth increases.

### 2.5. Sum-of-Least-Squares Assessment of Goodness-of-Fit

To evaluate the effectiveness of FNR-based correction across coverage levels, we quantified the similarity between PSMC trajectories using an independent sum-of-least-squares criterion. For each downsampled individual and each coverage level (5×–15×), we first identified the most optimal FNR value by minimizing either the Hausdorff distance [43] or the Fréchet distance [44] between the FNR-corrected trajectory and the corresponding high-coverage (non-downsampled) reference trajectory. This procedure yielded, for each downsample and for each distance metric, a single corrected curve with an optimal FNR value.

Subsequently, and independently of the optimization step, we computed the Sum of Squared Errors (SSE) between the best FNR-corrected trajectory and the high-coverage reference trajectory. SSE was therefore used exclusively as an evaluation metric and was not involved in the selection of the optimal FNR value. To calculate SSE, at each time point of the custom vector, we obtained the difference in effective population size (Ne) between the low-coverage and high-coverage trajectories, squared it, and summed it across all time points. This procedure was repeated independently for each downsampled coverage level and each individual, generating a set of SSE values corresponding to the best FNR correction at each depth. These SSE values were then used to assess how closely the optimally corrected trajectories approximated the high-coverage reference across coverage levels. Comparisons were based on direct inspection of SSE trends across depths and individuals.

## 3. Results

### 3.1. PSMC-FAC Enables Accurate FNR-Based Correction Across Species and Coverages

**Figure 2:**
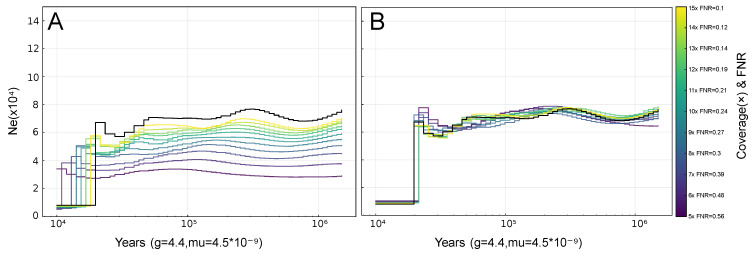
**Overlapping PSMC trajectories of sample Iberian grey wolf (SAMN43221691) before (A) and after (B) PSMC-FAC-based FNR correction.** Low coverage reduces heterozygote detection, shifting inferred coalescent events toward more recent times (leftward displacement) and lowering Ne estimates (downward displacement). FNR correction substantially restores trajectory concordance at moderate coverages (6–15×), whereas at very low coverages (<5×) correction becomes unreliable due to increased stochastic noise. Ne: population effective size.

**Figure A1:**
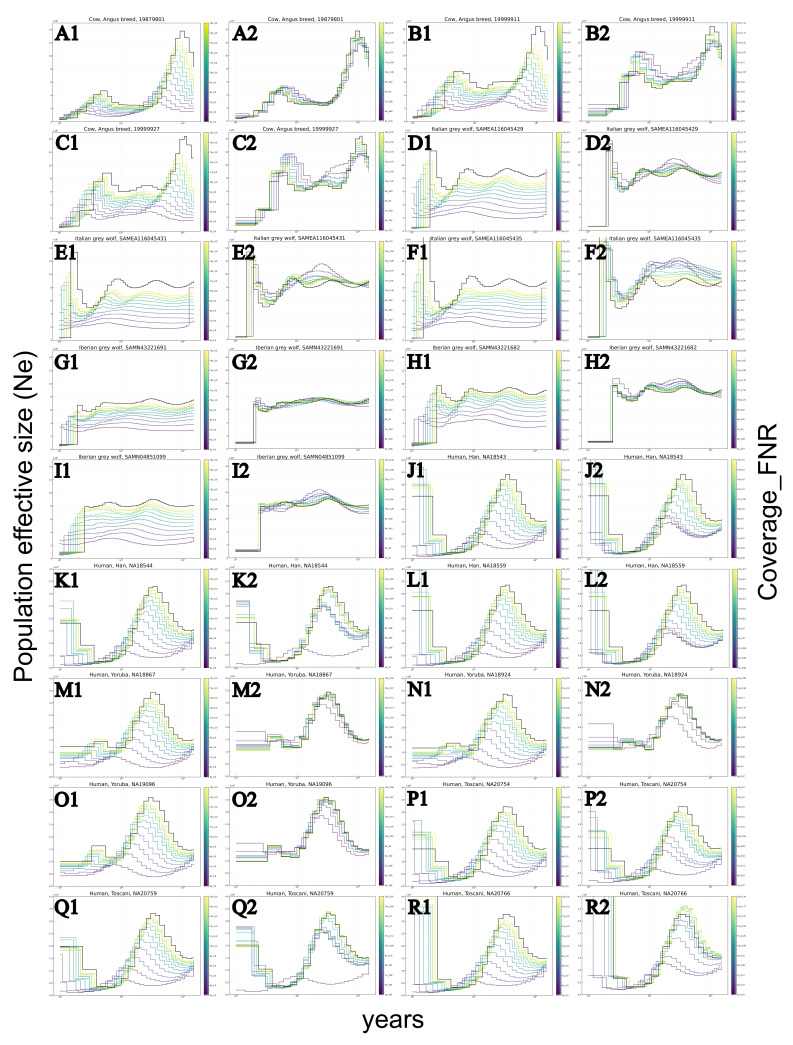
**PSMC demographic reconstructions using the original (20×) genome coverage and downsampled datasets ranging from 5× to 15×.** Results are shown without (1) and with (2) PSMC-FAC–based false negative rate (FNR) corrections. FNR corrections were estimated using time intervals between 10 kya and 1.5 Mya and applied accordingly to the reconstructions. Panels depict three cattle breeds (**A**–**C**), Italian grey wolves (**D**–**F**), Iberian grey wolves (**G**–**I**), and human genomes from China (Han; (**J**–**L**)), Nigeria (Yoruba; (**M**–**O**)), and Italy (Toscani; (**P**–**R**)).

Across all three species (cattle, wolves, and humans) and populations analyzed, PSMC-FAC produced mathematically consistent corrections of the false negative rate (FNR), substantially improving concordance between downsampled and original (20×) PSMC trajectories (Figure 2 and Figure A1, Table S1). As an example, at 10× coverage in a sample of Italian wolf, after an FNR correction considering the smallest Frechet distance, the SSE was reduced by 7.20 logarithmic units (see Table S1 for full results). After projection onto a common logarithmic time grid with a custom time vector and comparison to the corresponding high-coverage inference, FNR-corrected trajectories generally converged toward the reference curve across the evaluated temporal window (Figure A1) with the exception of the final abrupt population decreases, which may be attributed to the default time vector being used [21] (Figure A1).

Correction performance varied depending on both sequencing depth and demographic profile. In general, demographic reconstructions from genomes of higher coverages (10–15×) showed close agreement with the original trajectory after FNR correction. In contrast, medium-to-low-coverage datasets (5–6×) showed greater instability and reduced correction accuracy (Figure A1). At 5× coverage, corrected trajectories of gray wolves displayed slight increases in population size when the original 20× curve showed a slight decrease (Figure A1 panels D2, E2, F2, G2, H2, I2). This pattern is observed across all wolves and, although apparently paradoxical, is consistent with expectations from the variant-calling pipeline, since a minimum depth filter of 5 (-d 5) was applied during PSMC preprocessing, causing sites with depth lower than 5× to be ignored. In addition, the reference genome used for wolves (CanFam3.1) is known to exhibit relatively low heterozygosity [29].

PSMC-FAC was also able to adjust downsampled curves toward high-coverage trajectories in demographic scenarios characterized by abrupt population size changes, such as strong bottlenecks or rapid expansions (e.g., Figure A1 panels A1–A2, D1–D2, F1–F2, G1–G2). In such cases, corrected curves tended to smooth extreme transitions, suggesting that strong local curvature in inferred coalescent rates is especially sensitive to heterozygosity loss caused by downsampling.

### 3.2. Appropriate FNR-Based Correction Depends on Recent Demographic History

**Figure A2:**
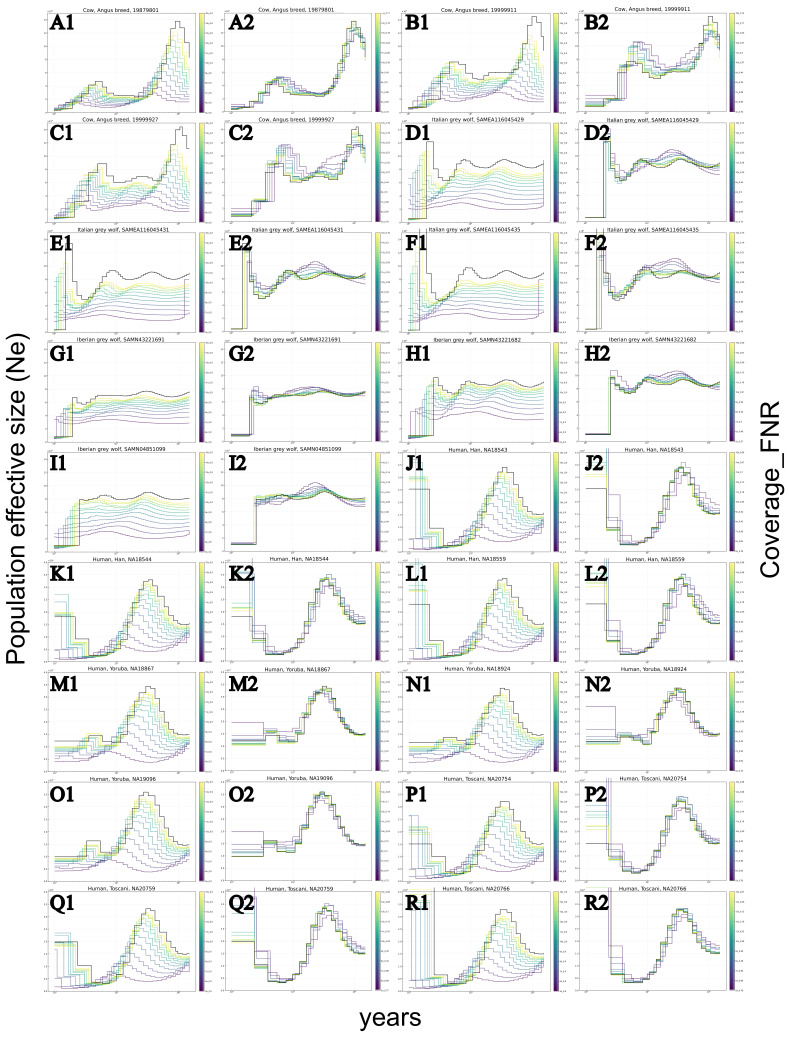
**PSMC demographic reconstructions using the original (20×) genome coverage and downsampled datasets ranging from 5× to 15×.** Results are shown without (1) and with (2) PSMC-FAC–based false negative rate (FNR) corrections. FNR corrections were estimated using time intervals between 50 kya and 1.5 Mya and applied accordingly to the reconstructions. Panels depict three cattle breeds (**A**–**C**), Italian grey wolves (**D**–**F**), Iberian grey wolves (**G**–**I**), and human genomes from China (Han; (**J**–**L**)), Nigeria (Yoruba; (**M**–**O**)), and Italy (Toscani; (**P**–**R**)).

**Figure A3:**
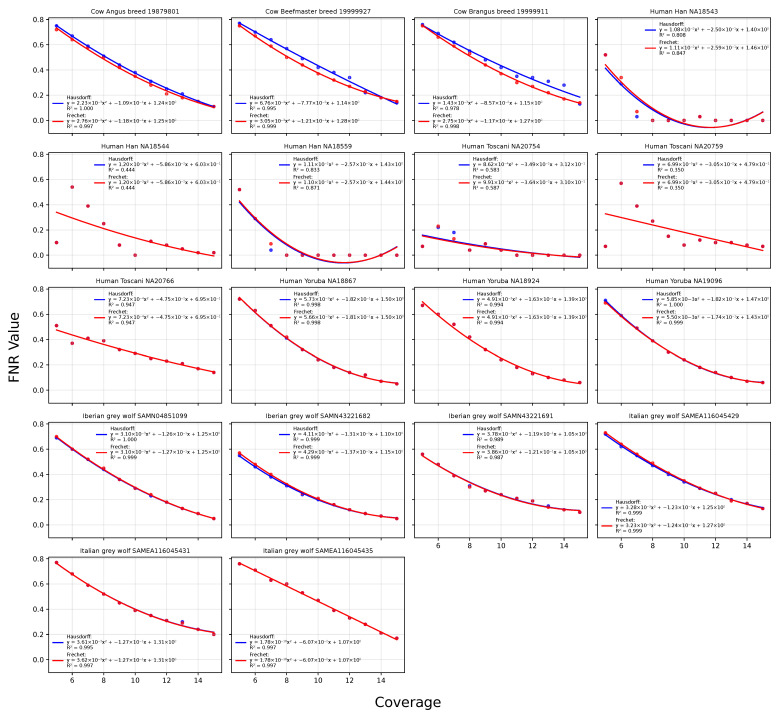
**Quadratic polynomial regressions describing the relationship between false negative rate (FNR; y-axis) and sequencing coverage (x-axis) for all downsampled individuals.** Polynomial models of degree 2 were fitted using both Hausdorff- and Fréchet-based optimization approaches. FNR values were estimated from comparisons performed across the time interval spanning 10 kya to 1.5 Mya.

Correction performance differed among human populations and appeared to depend on recent demographic history. While Yoruba (YRI) trajectories were consistently well corrected across coverages, with R^2^ between 0.994 and 1 (Figure A1 panels M–O, Figure A3), PSMC-FAC showed reduced performance for Han (CHB; Figure A1 panels J–L) and Toscani (TSI; Figure A1 panels P–R) genomes, with R^2^ between 0.444 and 0.871 (Han) and between 0.35 and 0.947 (Toscani) (Figure A2 and Figure A3). This discrepancy coincides with a pronounced population size increase inferred in non-African samples between  50–40 kya, where PSMC trajectories display a large, sharp elevation in effective population size during this interval. Because FNR optimization relies on minimizing Hausdorff and Fréchet distances between corrected and original trajectories [43,44], extreme local deviations can disproportionately influence the fitting procedure. If the last fragment of the demographic trajectory presents a sharp elevation, the algorithm will be biased to optimize correction parameters to better match this last maximum rather than the overall shape of the curve across the remaining temporal range.

To evaluate how PSMC-FAC adjusts downsampled curves to the original if the last peak is ignored, we repeated the FNR correction restricting the custom vector for the comparison to 50 kya–1.5 Mya for all genomes (Figure A2). This modification produced no substantial change for wolves or cattle (Figure A2 panels A–I), nor for Yoruba genomes (Figure A2 panels M2–O2), where the discrepancy was not found previously. In contrast, correction performance for Han (Figure A2 panels J2–L2) and Toscani (Figure A2 panels P2–R2) genomes improved markedly. When the recent sharp elevation in the curve was excluded from the fitting interval, corrected trajectories showed substantially better global concordance with the original high-coverage curves.

### 3.3. FNR Corrections Are Robust Across Diverse Demographic Histories

**Figure 3:**
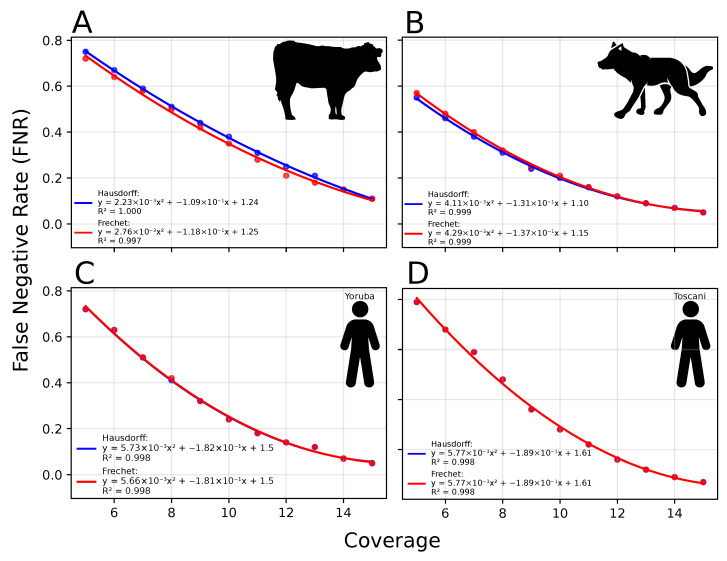
**Polynomial regressions of optimal FNR as a function of sequencing coverage for cattle (A), wolves (B), and humans (C,D).** Regressions were derived from PSMC-FAC-based FNR corrections using demographic trajectories between 10 kya and 1.5 mya (**A**–**C**) or between 50 kya and 1.5 mya (**D**), following the standard *psmc_plot.pl* output specifications. Strong and consistent correlations between FNR and coverage are observed across species and populations. Icons for cattle, wolves and humans were publicly available at the Noun Project www.thenounproject.com.

Across species and populations, optimization of FNR values produced highly concordant results between the two distance metrics used. When corrections were computed over the 10 kya–1.5 Mya window, Hausdorff and Fréchet distances were strongly correlated across the full panel (Figure 3 and Figure A3, Table S1), indicating that both metrics identified nearly identical optimal FNR values. The only clear exceptions were the non-African human populations (Han and Toscani), consistent with the influence of the pronounced recent-time Ne peak described above. For all genomes, the relationship between sequencing coverage and inferred optimal FNR followed a highly regular polynomial trend. In every case, the fitted models yielded ($R^{2}>0.99$), demonstrating a very close relationship between depth of coverage and the magnitude of FNR correction required. This pattern was consistent across cattle ($R^{2}\in \;\left[0.978,\;1\right]$), wolves ($R^{2}\in \;\left[0.978,\;1\right]$), and humans ($R^{2}\in \;\left[0.994,\;1\right]$) (Figure A3), despite their distinct demographic histories and differences in reference genomes and time interval parametrization. Importantly, Hausdorff and Fréchet distances performed nearly equivalently in identifying optimal FNR values. Differences in the selected FNR were minimal and resulted in only marginal variation in goodness-of-fit statistics for the coverage–FNR regression curves. Thus, correction accuracy does not depend strongly on the specific choice of distance metric, reinforcing the robustness of the PSMC-FAC optimization framework.

**Figure A4:**
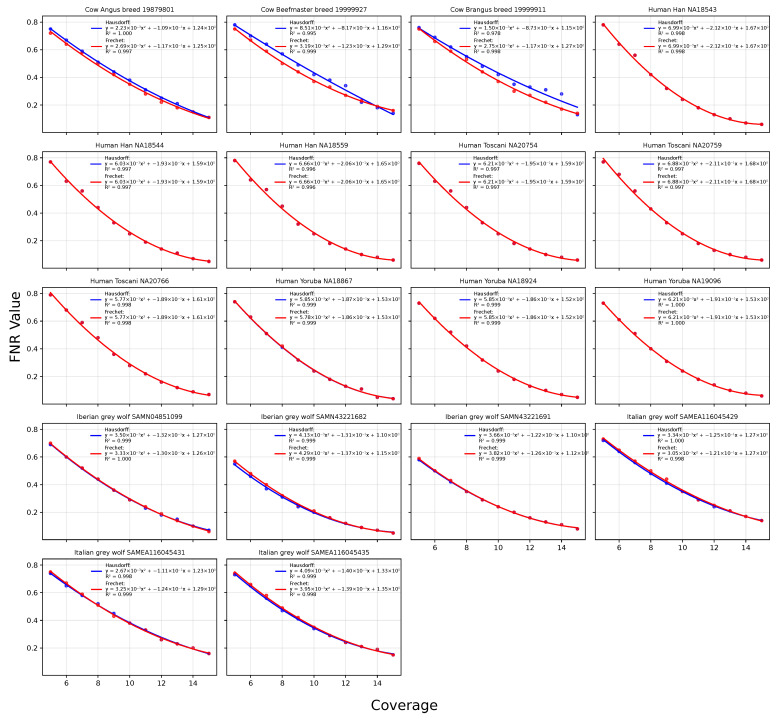
**Quadratic polynomial regressions describing the relationship between false negative rate (FNR; y-axis) and sequencing coverage (x-axis) for all downsampled individuals.** Polynomial models of degree 2 were fitted using both Hausdorff- and Fréchet-based optimization approaches. FNR values were estimated from comparisons performed across the time interval spanning 50 kya to 1.5 Mya.

After restricting the optimization window to 50 kya–1.5 Mya and re-estimating FNR values, the previously observed discrepancy in non-African human populations largely disappeared (Figure A4; Table S2). Han and Toscani genomes then showed near-perfect correlations between genomic coverage and optimal FNR, comparable to those observed in Yoruba, wolves, and cattle. In contrast, only negligible changes were observed for the other genomes, whose trajectories lack the extreme recent-time Ne increase. These results strongly suggest that deviations observed in specific populations were not due to intrinsic limitations of the correction framework but rather to localized, high-amplitude features in the recent portion of the trajectory that disproportionately influenced distance-based optimization. Once these features were excluded, the coverage–FNR relationship became uniformly stable and highly predictable across all species and populations analyzed, making it possible to use it as a mathematical tool to predict the FNR needed for other coverages.

**Figure A5:**
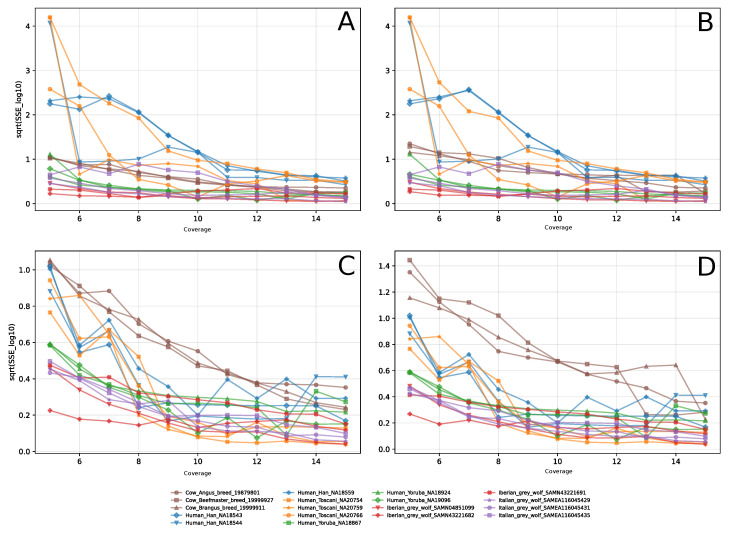
**Log-scaled sum of squared errors per coverage and sample of every FNR calculation according to optimal Fréchet (A,C) or Hausdorff (B,D) distances.** FNR values were estimated from comparisons performed across the time interval spanning 10 kya to 1.5 Mya (panels (**A**,**B**)) or 50 kya to 1.5 Mya (panels (**C**,**D**)).

Although FNR optimization behaved consistently within species and populations, the shape of the polynomial relationship between sequencing coverage and optimal FNR differed among taxa (Figure A3, Figure A4 and Figure A5). These differences highlight the distinct demographic histories and genome-wide heterozygosity levels observed across cattle, wolves, and humans (Table A1). Thus, while the coverage–FNR relationship is highly predictable within a population (*R^2^* > 0.99 in all cases), it is not universally transferable across populations with divergent demographic backgrounds, so running a PSMC-FAC pipeline per species or per population is highly advisable.

## 4. Discussion

In conservation and population genomics studies, there is an inherent trade-off between sequencing depth and sampling breadth [45,46]. High-coverage whole-genome sequencing remains costly, and generating large numbers of genomes at  20× depth is often financially prohibitive for many research groups. Conversely, sequencing a small number of individuals at high coverage is insufficient to capture the demographic heterogeneity of structured populations [47]. Although low-coverage genomes are frequently considered unsuitable for demographic inference, previous work has shown that meaningful demographic signals can be retained even under reduced representation approaches such as RAD sequencing under specific conditions [48]. Nadachowska-Brzyska et al. [9] demonstrated that PSMC performance declines substantially below approximately 10× coverage and recommended higher depths for stable inference. Building on these observations, we show that low- and medium-coverage genomes can still yield informative demographic inference when the loss of heterozygosity is corrected quantitatively using a False Negative Rate (FNR) factor. By deriving population-specific calibration curves linking FNR and sequencing coverage, the PSMC-FAC framework reduces uncertainty and allows demographic analyses to rely on a combination of high- and low-coverage genomes, thereby lowering overall sequencing costs for sound population-based demographic inference.

### 4.1. FNR Correction in Low- and Mid-Depth Genomes: Reference Genome Effect

At low coverage (e.g.,  5×), error rates remain substantial and FNR correction cannot fully compensate for stochastic loss of information. In contrast, between approximately 8× and 15× coverage, corrected trajectories converge closely toward high-coverage references, substantially expanding the practical utility of medium-depth sequencing data. The reduced performance observed at very low coverage reflects limitations of the variant-calling pipeline rather than a failure of the correction framework itself. During the PSMC preprocessing, a minimum depth threshold of five reads is applied (parameter -d 5). When mean coverage is  5×, a substantial proportion of true heterozygous sites inevitably falls below this threshold and is therefore excluded. These sites are subsequently treated as homozygous reference during consensus generation, leading to systematic underestimation of heterozygosity. Because PSMC infers demographic history from the spatial distribution of heterozygous tracts along the genome, such undercalling directly alters the inferred coalescent rate. The resulting distortion is therefore not random noise but a predictable shift in trajectory shape, affecting inferred effective population size and potentially generating spurious demographic features. Similar coverage-dependent biases have been documented previously, particularly by Nadachowska-Brzyska et al. [9], who showed that decreasing depth disproportionately impairs heterozygote detection and destabilizes demographic inference. Our results are consistent with these observations. Importantly, while PSMC-FAC corrects for heterozygosity loss attributable to false negatives, at sufficiently low coverage both stochastic and systematic variation introduce an irreducible source of error. This is reflected in the elevated sum-of-squared errors observed even at the optimal FNR value at the 5× downsamplings. Thus, while the framework substantially mitigates coverage-related bias, it cannot completely overcome the intrinsic limitations of very shallow sequencing data.

These effects may become particularly pronounced when the reference genome does not adequately represent the genome-wide diversity of the population under study. For example, the human reference assembly (hg38/GRCh38) was constructed from a limited number of individuals and does not capture global human genetic diversity [49,50]. Similarly, the domestic dog reference genome, although widely used in canid studies, does not fully represent the diversity present across wolf and dog populations [51,52]. Under such circumstances, mapping bias toward the reference allele can further exacerbate heterozygote undercalling, amplifying the apparent loss of heterozygosity at low coverage. Consequently, the accuracy of FNR correction depends not only on sequencing depth but also on the appropriateness of the chosen reference genome. This highlights the importance of considering reference bias when interpreting demographic reconstructions derived from low-coverage data. As a possible solution to this caveat, we propose the use of population-matched or pan-genome references, graph-based genome representations, or the construction of pseudodiploid consensus sequences, all of which can reduce mapping bias and improve heterozygote detection in diverse populations.

### 4.2. Effects of Biases Introduced by PSMC Assumptions on Optimal FNR Calculation

The pronounced peak observed in non-African human populations between approximately 50 and 40 thousand years ago provides a revealing example of how features of a PSMC trajectory can influence FNR optimization. When a broad temporal window starting at 10 kya was used for distance minimization, this sharp recent-time elevation disproportionately influenced both Fréchet and Hausdorff distance metrics. As a consequence, optimization tended to favor FNR values that improved agreement around this localized feature rather than across the trajectory as a whole. Similar peaks were already noted in the original PSMC publication by Li and Durbin (2011) [5], and their biological interpretation has remained controversial. More recent work has demonstrated that population structure alone can generate such sharp biasing peaks in SMC-based inference due to coalescent signatures (Nieto et al. 2025, in press) [20]. Tournebize et al. (2025) [53] further demonstrated that structure and admixture can produce nearly indistinguishable coalescent patterns. Admixture with Neanderthals in non-African populations between approximately 50 and 43 kya remains a plausible biological contributor to this pattern, as interspecific gene flow is known to generate transient increases in inferred effective population size under SMC-based frameworks [27,54]. However, because these peaks are also sensitive to the temporal discretization parameters used in PSMC [21], this feature is interpreted conservatively as a methodological artefact lacking straightforward demographic interpretation. When the optimization window was restricted to exclude this recent interval (starting at 50 kya), FNR–coverage relationships stabilized markedly across Toscani and Han populations, whereas little change was observed for other populations and species. This indicates that the instability did not originate from the correction framework itself but rather from biases present in the reference trajectory. In practice, adjustment of PSMC hyperparameters and careful selection of the temporal window should be performed prior to FNR optimization in order to reduce artefact-driven bias in the reference curve.

This observation leads to a central conceptual clarification: PSMC-FAC optimizes trajectory similarity rather than recovering true demography. The method minimizes geometric distance between a low-coverage trajectory and a chosen high-coverage reference; consequently, any artefacts contained in the reference trajectory are inherently propagated through the correction process. If the reference curve contains artefacts arising from structure [20], background selection [17], time discretization [21], inclusion of functional genomic elements in the problem sequence [16], or model misspecification [24], the FNR correction will reproduce them. PSMC-FAC therefore corrects coverage-dependent heterozygosity loss but does not address the theoretical or methodological limitations intrinsic to the Sequentially Markovian Coalescent (SMC) framework. Sellinger et al. (2021) [16] demonstrated that SMC-based methods have intrinsic convergence properties sensitive to mutation–recombination ratio assumptions, potentially limiting applicability in some populations. The present framework does not resolve such limitations; instead, it isolates and corrects one specific and pervasive source of bias: false-negative heterozygous calls caused by limited sequencing depth.

### 4.3. Polynomial Relationship Between Coverage and Optimal FNR

The strong and consistent polynomial relationship observed between sequencing coverage and optimal FNR indicates that heterozygosity loss scales predictably with depth under stable analytical conditions. Within individual populations, this relationship is highly regular, allowing coverage-dependent correction curves to be estimated with high confidence. However, the shape and parameters of these polynomial functions differ across species and populations, reflecting variation in genome-wide heterozygosity levels, recombination landscapes, and demographic history.

Demographic features themselves also influence optimization behavior. Sharp events, such as rapid bottlenecks or expansions, generate regions of high local curvature in PSMC trajectories. These features can disproportionately influence distance-based comparisons and, consequently, shift the inferred optimal FNR values. Thus, FNR estimation is determined not only by sequencing coverage but also by the geometric properties of the underlying demographic trajectory. The combined use of Hausdorff and Fréchet distances [43,44] provides complementary perspectives on this problem. The Hausdorff distance is sensitive to localized maximum deviations, whereas the Fréchet distance captures overall trajectory similarity while preserving temporal ordering. Together, these metrics offer a robust framework for estimating coverage-dependent correction parameters while accounting for variation in demographic trajectory shape.

Furthermore, the assumption that the coverage–FNR relationship derived from a single individual is transferable to other individuals within the same population is not explicitly tested here. While consistency across populations suggests robustness, inter-individual variation in heterozygosity patterns or sequencing characteristics may affect the generalizability of the fitted correction functions and should be considered when applying the method.

### 4.4. Empirical Applications and Future Implications

Several alternative strategies have been proposed to extract demographic information from low-coverage genomic data, including genotype likelihood-based approaches (e.g., ANGSD), imputation-based methods, and variant re-calling pipelines using tools such as GATK. These approaches aim to improve heterozygote detection directly at the variant-calling stage, thereby mitigating coverage-related biases. In contrast, PSMC-FAC operates downstream of variant calling, correcting the resulting demographic trajectories rather than the underlying genotype calls. As such, it is complementary to these methods and can, in principle, be applied on top of improved genotype likelihood or imputation frameworks. Additionally, multi-sample SMC-based methods such as MSMC and SMC++ partially alleviate coverage limitations by leveraging information across genomes; however, they remain sensitive to heterozygosity undercalling. Extending PSMC-FAC as a preprocessing or calibration layer for these approaches represents a promising direction for future work.

In practical terms, this framework enables demographic analyses in conservation and population genomics contexts where sequencing large numbers of individuals at high coverage is economically unfeasible. By calibrating coverage-dependent bias using a high-coverage reference genome, additional individuals sequenced at moderate depth can be incorporated into demographic analyses without relying on subjective or visually determined FNR adjustments. This substantially expands the range of sampling designs compatible with PSMC-based inference while maintaining methodological consistency across individuals.

In this context, the high-coverage genome should be interpreted as an empirical calibration reference rather than a ground truth representation of the population’s demographic history. The validity of the correction therefore depends on how well this reference captures the underlying coalescent signal shared across individuals. When this assumption is reasonably met, PSMC-FAC enables consistent scaling of low-coverage trajectories; however, deviations due to population substructure, admixture, or technical artifacts in the reference may limit transferability and should be carefully considered when interpreting results.

These results should also be interpreted in light of the known temporal limits of PSMC inference. Patton et al. (2019) [55] demonstrated that SMC-based methods achieve their highest resolution at intermediate timescales, whereas accuracy declines for very recent demographic events. More recently, Peede et al. [56] emphasized that temporal resolution depends strongly on the distribution of coalescent and recombination events along the genome. To accommodate these constraints, PSMC-FAC allows the specification of custom temporal windows during FNR optimization, enabling the exclusion of intervals known to contain unstable or artefactual signals. Consequently, low-coverage genomes corrected using PSMC-FAC should not be interpreted at very recent or fine-scale temporal resolutions. Still, they can provide robust information for intermediate and deep-time demographic inference where PSMC performs most reliably.

An important consideration for practical application is the transferability of the optimized FNR coefficients across individuals. Within a given population, we expect these coefficients to generalize well, as individuals typically share similar demographic histories and therefore comparable PSMC trajectories, provided that sequencing characteristics are consistent. However, transferability across populations is likely to depend on the similarity of their underlying demographic histories, and population-specific recalibration may be required when these differ substantially. Additionally, individual-level deviations due to substructure or admixture are not expected to be masked by the correction, but rather preserved in the inferred trajectories, suggesting that the method can be applied without obscuring biologically meaningful variation.

PSMC-FAC provides a reproducible and mathematically grounded procedure for calibrating coverage-dependent bias. By replacing subjective visual adjustment of FNR values with distance-based optimization metrics, the framework improves reproducibility and allows explicit quantification of correction performance. Although currently implemented as a correction layer for PSMC trajectories, the underlying conceptual approach could potentially be extended to other SMC-based methods that rely on heterozygosity patterns, provided that suitable high-coverage reference data are available.

A noticeable limitation of PSMC-FAC is that it specifically targets coverage-dependent bias and does not correct for other sources of systematic error inherent to PSMC inference or the high-coverage reference data itself (such as mapping biases, correct definition of time vector, appropriate window coordinates for the plot, etc.). As the method optimizes similarity to a high-coverage reference trajectory, any biases present in that reference may be propagated into the corrected results. Therefore, careful validation of the high-coverage genome is essential, including consistency across individuals, absence of technical artifacts, and concordance with prior studies. Additionally, deviations from the expected FNR–coverage relationship, such as unstable fits or poor model performance, may serve as practical diagnostic indicators of underlying biases not attributable to sequencing depth, and should be interpreted with caution.

Overall, these results demonstrate that low-coverage genomes are not inherently unsuitable for demographic inference. When appropriately calibrated, they can approximate high-coverage demographic trajectories within predictable limits. By correcting the component of distortion attributable specifically to sequencing depth, PSMC-FAC lowers the practical barrier to demographic reconstruction and enables broader sampling strategies, making population-scale PSMC analyses more accessible to laboratories for which large-scale high-coverage sequencing remains economically unfeasible.

## 5. Conclusions

This study introduces PSMC-FAC, an automated and reproducible framework that corrects coverage-dependent biases in demographic reconstructions derived from low-coverage genomes, validated across three evolutionarily and demographically distinct species. By replacing subjective visual adjustments with objective, distance-based optimization, the method enables consistent estimation of false-negative rate (FNR) corrections and substantially improves concordance between low- and high-coverage demographic trajectories across diverse species and demographic scenarios.

Our results show that low- and medium-coverage genomes can provide reliable demographic information when appropriately calibrated using population-specific correction curves. Although the framework does not address inherent theoretical limitations of Sequentially Markovian Coalescent models, it effectively corrects a major practical source of bias associated with sequencing depth. By reducing reliance on high-coverage datasets, PSMC-FAC lowers sequencing cost constraints, supports broader sampling designs, and expands the practical applicability of demographic inference in evolutionary biology, conservation genomics, and comparative population studies.

## Data Availability

PSMC-FAC, its scripts and some example data used in this work can be found at https://github.com/franiiss/PSMC-FAC, accessed on 6 April 2026.

## Supplementary Materials

The following supporting information can be downloaded at: https://www.mdpi.com/article/10.3390/biology15080631/s1, Table S1: Optimal FNR value per coverage and sample according to Hausdorff and Frechet distances. FNR was calculated in PSMC files with a custom vector spanning between 10 kya and 1.5 Mya; Table S2: Optimal FNR value per coverage and sample according to Hausdorff and Frechet distances. FNR was calculated in PSMC files with a custom vector spanning between 50 kya and 1.5 Mya.

## Abbreviations

    The following abbreviations are used in this manuscript:

PSMC	Pairwise Sequentially Markovian Coalescent
FNR	False-Negative Rate
PSMC-FAC	PSMC False-Negative Rate Automatized Correction
WGS	Whole-Genome Sequencing
Ne	Effective Population Size
TMRCA	Time to the Most Recent Common Ancestor
ARG	Ancestral Recombination Graph
HMM	Hidden Markov Model
SMC	Sequentially Markov Coalescent
MSMC	Multiple Sequentially Markovian Coalescent
RAD	Restriction Site Associated DNA
BAM	Binary Alignment/Map format
CRAM	Compressed Reference-oriented Alignment Map format
VCF	Variant Call Format
PSMCFA	PSMC FASTA-like input format
PCR	Polymerase Chain Reaction
SSE	Sum of Squared Errors

CHB	Han Chinese in Beijing, China (1000 Genomes Project population)
YRI	Yoruba in Ibadan, Nigeria (1000 Genomes Project population)
TSI	Toscani in Italia (1000 Genomes Project population)
1000GP	1000 Genomes Project
ARS-UCD1.2	Agricultural Research Service, United States Department of Agriculture
GRCh38	Genome Reference Consortium Human Build 38
Hg38	Human Genome version 38
CanFam3.1	Dog Reference Genome Assembly Version 3.1
BosTau9	Cattle Reference Genome Assembly Version 9
kya	Thousand Years Ago
Mya	Million Years Ago
DNA	Deoxyribonucleic Acid
$R^{2}$	Coefficient of Determination

## Appendix A. Computational Workflow for PSMC Processing and FNR Estimation Using PSMC-FAC

### Appendix A.1. Preparation of PSMC Input Files

PSMC-FAC is a bioinformatic pipeline designed to correct for False Negative Rate (FNR) reliably through the use of mathematical distances in a plane between a Pairwise Sequential Markovian Coalescent (PSMC) demographic curve of a high-coverage genome and PSMC curves of downsamples of the same genome at different low-to-medium coverages. The goal is to get a trustable extrapolation of coverages with FNR, which allows researchers to run multi-sample PSMC graphs of a population without having to spend enormous resources on whole-genome sequencing for multiple samples. PSMC-FAC needs only to have one sample at high coverage (>18X) which will be downsampled to several coverages. After FNR is corrected for every coverage, a FNR vs coverage plot relationship will be drawn and a polynomial regression can be calculated, after which low-to-medium-coverage samples can be used. This procedure avoids using the same genome to construct a bootstrapped individual-level PSMC plot [5] and allows the use of entire populations, therefore allowing visualization of population structure and diverse demographic trajectories.

The initial processing steps follow the standard workflow described by [5] and outlined in their github page (https://github.com/lh3/psmc, accessed on 10 January 2026). Prior to running the pipeline, a high-coverage genome (typically > 18×) must be aligned to a reference genome in FASTA format (ref.fa) or obtained as an aligned BAM file. PCR duplicates and spurious alignments should be removed before further analysis. Then, the .bam file is converted into variant call format (VCF) with bcftoolsv1.13 mpileup and call commands and later transformed into consensus FASTQ sequences as in [5]:

bcftools mpileup -C50 -f ref.fa -Ou highcovgenome.bam | bcftools call -c -Oz -o hcgenome.vcf.gz (step 1)

bcftools view hcgenome.vcf.gz | vcfutils.pl vcf2fq -d 5 -D 2*AverageCoverage hcgenome.fq (step 2)

In step (2), sites with depth below 5× are excluded, and positions exceeding twice the average genomic coverage are removed to reduce artifacts associated with duplicated regions and abnormal read depth.

Consensus FASTQ files are then converted into PSMC input format (PSMCFA) applying a minimum phred-scale base quality threshold of 20 using:

psmc/fq2psmcfa -q20 hcgenome.fq > hcgenome.psmcfa (step 3)

Then, demographic inference is performed using PSMC:

psmc -N20 -t10 -r5 -p “time_vector” -o hcgenome.psmc hcgenome.psmcfa (step 4)

where the time interval configuration (time_vector) is species-specific, as described in the Dataset Preparation Subsection of the Materials and Methods. Although many studies use the default time_vector described in [5], this was designed originally for human populations.

For non-human populations, the use of a custom-made time vector according to the specificities of the demography of the target population is recommended. In particular, adjusting the interval configuration can reduce artefacts associated with recent-time population size increases, as discussed in [20,21].

To construct the FNR–coverage correction curve, the high-coverage genome is downsampled to multiple target depths. In practice, approximately 8–10 coverage levels are sufficient to accurately estimate the coverage–FNR relationship. Downsampling is performed using samtools view -s, where the sampling fraction corresponds to the ratio between target and original coverage. For example, reducing a 20× genome to approximately 5× requires retaining 25% of reads:

samtools view -s 0.25 -b 20xgenome.bam > 5xgenome.bam (step 5)

This procedure should be repeated for each desired coverage level. After downsampling, steps (1)–(4) are repeated for every generated BAM file to obtain a set of PSMC trajectories spanning multiple coverages. These trajectories form the basis for FNR estimation and subsequent correction modeling within PSMC-FAC.

### Appendix A.2. Usage of PSMC-FAC

#### Appendix A.2.1. Summary

PSMC-FAC automatizes the process of finding the optimal FNR-corrected curve per downsampled genome using either the Hausdorff or Fréchet distances. For each downsampled genome, PSMC-FAC evaluates a discrete grid of candidate FNR values spanning the interval [0, 0.99] in increments of 0.01. Application of each candidate FNR produces a corrected PSMC trajectory, resulting in a set of 100 demographic curves per downsampled dataset. These corrected trajectories are compared against the corresponding high-coverage reference trajectory obtained from the same individual. Prior to comparison, all trajectories are projected onto a common logarithmically spaced temporal grid to ensure point-wise correspondence across time while preserving the stepwise structure inherent to PSMC inference. Each trajectory is therefore represented as an ordered sequence of coordinates in two-dimensional space:

$$P={\left\{\left(t_{i},N_{e,i}^{\mathrm{ref}}\right)\right\}}_{i=1}^{n},\;Q_{f}={\left\{\left(t_{i},N_{e,i}^{\left(f\right)}\right)\right\}}_{i=1}^{n}$$

According to user specifications, PSMC-FAC evaluates all 100 FNR-corrected trajectories by computing either the discrete Fréchet distance or the Hausdorff distance between each corrected curve and the corresponding high-coverage reference trajectory. The selected metric is used to systematically quantify the geometric discrepancy between trajectories across the full range of tested FNR values.

#### Appendix A.2.2. User Manual

PSMC-FAC is a unified Python workflow designed to evaluate false-negative-rate (FNR) corrections for Pairwise Sequentially Markovian Coalescent (PSMC) trajectories inferred from high- and reduced-coverage genomes. This software integrates five tasks within a single pipeline: (1) extraction of FNR-corrected demographic trajectories from PSMC output files, (2) calculation of Hausdorff and Fréchet distances between downsampled and reference trajectories, (3) computation of residual error metrics based on the sum of squared errors (SSE), (4) estimation of optimal FNR values across coverage levels, (5) and visualization of both polynomial correction curves and corrected demographic trajectories. This implementation allows all analyses to be run from a single command-line interface.

The workflow assumes that each reference (or high-coverage) genome and its associated downsampled (lower-coverage) genomes are stored within the same directory. The program can be executed either on a root directory containing multiple subdirectories with samples of the same species or on a single directory containing only one set of reference and less coverage .psmc files. In both cases, the script recursively scans the supplied root path and processes every directory in which PSMC files are present.

The first step consists of extracting the final iteration of each .psmc file and generating FNR-corrected demographic trajectories. For each input genome, the script reads the last PSMC iteration, recovers the discretized time intervals and corresponding coalescent parameters, and recomputes effective population sizes after applying a sequence of FNR values ranging from a minimum to a maximum value in increments of 0.01 (default values are 0 and 0.99, considering all range of possible FNR values). Each corrected trajectory is then resampled over a custom vector of n logarithmically spaced years using the piecewise-constant logic as in the original PSMC implementation that can be specified by the user. This allows the user to select the range of time in which the inference is considered to get the best FNR correction. This step can optionally (recommended) be exported as one TSV file per .psmc input, in which the first column would be the years and the following columns all the Ne values considering the FNR correction specified in the column name.

The second step compares each FNR-corrected curve from each downsampled (or less coverage) trajectory against its corresponding reference trajectory. For every tested FNR value, the program calculates the symmetric Hausdorff distance and the discrete Fréchet distance between the corrected trajectory and the reference. These distances are computed after transforming the coordinates according to a user-specified plotting scale. Three modes are currently supported: a fully linear scale, a fully logarithmic scale, and a PSMC-like scale in which time is represented in log10 units and effective population size is multiplied by $10^{4}$. The latter is the default option because it mimics the standard visual representation used in the original PSMC plot script by Li and Durbin (2011) [5] (psmc_plot.pl). In parallel, the script computes both the raw SSE and the SSE in log10 space, allowing the user to quantify residual discrepancies after correction independently of the metric used to select the best FNR. Note that the best FNR correction may have still a high SSE, specially for low-coverage samples (less than 5x).

The output of this stage is written to a large tab-delimited file containing one row per sample, coverage, and tested FNR value. This file includes the sample identifier, the coverage value, the tested FNR, the Hausdorff distance, the Fréchet distance, the SSE, the log10(SSE), and the coordinate scaling mode used for the distance calculation. This table constitutes the core analytical output of the pipeline because it records the full relationship between correction value and trajectory similarity at every downsampling level.

A second summary file is then generated by selecting, for each sample and coverage level, the row with minimum Hausdorff distance and the row with minimum Fréchet distance. This reduced table therefore contains one entry per sample and coverage and reports the optimal FNR according to each distance metric together with the associated residual SSE values. This output is intended to facilitate direct comparison across samples and coverage levels and can be used for downstream visualization or model fitting.

An optional curve-fitting step estimates how the best FNR varies with sequencing coverage. For each sample, the script extracts the FNR values that minimize Hausdorff distance and the FNR values that minimize Fréchet distance, and it attempts to fit a quadratic polynomial of the form $y=ax^{2}+bx+c$, where x is coverage and y is the optimal FNR. Quadratic fitting is only performed when at least three coverage levels are available for a given sample, since fewer points do not support a second-degree polynomial fit. When fewer than three coverages are available, the program reports that the curve was skipped. The corresponding fitted equations are written to a text file, and the selected optimal points are also exported in separate CSV files for Hausdorff and Fréchet.

A second optional (recommended) visualization step plots the inferred demographic trajectories. For each sample, the script generates a multi-panel PDF in which each panel corresponds to one downsampled genome. Within each panel, the reference trajectory along with the uncorrected downsampled trajectory (FNR = 0), the trajectory corrected with the best Hausdorff-derived FNR, and the trajectory corrected with the best Fréchet-derived FNR are displayed in three different panels. These plots provide a direct visual assessment of whether the selected corrections recover the shape and temporal position of the reference demographic history.

The program supports two alternative ways of defining the reference genomes. The first is to provide the reference filenames explicitly on the command line using *–base_files*. In this mode, all other PSMC files are treated as downsampled genomes, and their coverage values are inferred from the naming convention sample.<coverage>.psmc. The second is to provide a coverage table with the argument –coverage_table. In that mode, the table must contain all .psmc files detected under the input root directory and must specify, for each file, either a numeric coverage value or the tag baseline. This second mode is particularly useful when the file naming convention does not encode coverage directly or when coverage values need to be set manually.

The way to call PSMC-FAC is as follows:

Python PSMC-FAC.py –root /path/to/files/directory –output /path/to/output –base_files list_of_filenames –coverage_table cov.tsv –mu mut_rate_value –g gen_ time –svalue 100 –FNR_min 0 –FNR_max 0.99 –tmin 1e4 –tmax 1.5e6 –n timepoints –distance_scale [psmc, linear, loglog] –write_psmc_tsv –no_curves –no_trajectory _plots

Options:

–root: Root directory containing one or more directories of .psmc files.

–outdir: Output directory in which all generated files are written.

–base_files: List of filenames corresponding to the reference genomes. This argument is required when no coverage table is supplied.

–coverage_table: Optional tab-delimited table with columns psmc_file and coverage. If provided, it may define baseline files using the label baseline.

–mu: Per-generation mutation rate used to transform coalescent parameters into demographic trajectories.

–g: Generation time used to convert generations into years.

–svalue: Parameter -s used in PSMC. Default is 100.

–FNR_min: Minimum FNR value to test. Default is 0.

–FNR_max: Maximum FNR value to test. Default is 0.99.

–tmin: Minimum year value used to define the custom time vector. Default is  1 × 10^4^.

–tmax: Maximum year value used to define the custom time vector. Default is  1.5 × 10^6^.

–n_timepoints: Number of points used in the custom time vector. Default is 60.

–distance_scale: Coordinate transformation used for Hausdorff and Fréchet distances; allowed values are psmc, linear, and loglog.

–write_psmc_tsv: Optional flag instructing the script to write one TSV per PSMC file containing all FNR-corrected trajectories.

–no_curves: Optional flag to disable polynomial fitting and the associated outputs.

–no_trajectory_plots: Optional flag to disable corrected versus uncorrected trajectory plots.

Several practical considerations should be kept in mind when using PSMC-FAC. The software processes PSMC files at the level of the terminal input directories identified under the user-specified root path. Within each such directory, all downsampled genomes are evaluated against the reference genome or genomes present in that same folder. Accordingly, reference and downsampled (lower-coverage) PSMC files must be placed together in the same final directory level for a comparison to be performed. When a coverage table is supplied, it must match exactly the set of .psmc files found under the input root; missing or extra entries cause the program to stop with an error. Polynomial fitting should only be interpreted when a sufficient number of coverage levels are available, since sparse sampling makes the estimated coverage–FNR relationship unstable. Finally, the choice of distance scale may affect the selected optimal FNR values because Hausdorff and Fréchet are geometric metrics defined on transformed coordinates; the default PSMC-like scale is therefore recommended when the goal is to mimic the visual comparison typically used in manual correction.

### Appendix A.3. Plotting Other Low-Coverage Genomes According to PSMC-FAC-Assisted FNR Correction

Once FNR values have been assigned to each genome, corrected trajectories can be visualized using the standard plotting utilities provided in the original PSMC package. FNR values are supplied through the -M option of psmc_plot.pl, which specifies the correction applied to each sample. For example, given three genomes with different sequencing coverages (genome1 = 20×, genome2 = 5.73×, genome3 = 7.5×) and corresponding FNR values inferred from the regression model (0, 0.53, and 0.22, respectively), plotting is performed as follows:

psmc_plot.pl -M “genome1=0,genome2=0.53,genome3=0.22” prefix genome1.psmc genome2.psmc genome3.psmc (step 6)

Following these steps, a bootstrapped PSMC plot can be generated using multiple genomes with heterogeneous sequencing coverages while applying coverage-specific FNR corrections.

## Appendix B. Appendix Figures

**Table A1:** Summary of samples included in this study, comprising individuals from three species (Bos taurus, Canis lupus, and Homo sapiens), representing multiple populations and breeds. Data sources are as follows: (A) [57]; (B) [52]; (C) [58]; (D) [32].

Common Name	Scientific Name	Population	Sample Number	Coverage	Heterozygosity	Source
Cow	*Bos taurus*	Angus breed	19879801	19.18X	$2.38\;\times \;10^{-3}$	(A)
		Brangus breed	19999911	39.47X	$3.98\;\times \;10^{-3}$	(A)
		Beefmaster breed	19999927	31.11X	$3.92\;\times \;10^{-3}$	(A)
Grey wolf	*Canis lupus*	*C. l. italicus* (Italian wolf)	SAMEA116045429	23.67X	$1.68\;\times \;10^{-3}$	(B)
			SAMEA116045431	27.19X	$1.48\;\times \;10^{-3}$	(B)
			SAMEA116045435	26.08X	$1.44\;\times \;10^{-3}$	(B)
		*C. l. signatus* (Iberian wolf)	SAMN43221691	20.42X	$1.87\;\times \;10^{-3}$	(C)
			SAMN43221682	19.03X	$1.81\;\times \;10^{-3}$	(C)
			SAMN04851099	18.08X	$1.88\;\times \;10^{-3}$	(C)
Human	*H. sapiens*	Han Chinese (CHB)	NA18543	29.59X	$1.00\;\times \;10^{-3}$	(D)
			NA18544	29.53X	$9.82\;\times \;10^{-4}$	(D)
			NA18559	33.34X	$9.89\;\times \;10^{-4}$	(D)
		Yoruba, Nigeria (YRI)	NA18867	30.18X	$1.32\;\times \;10^{-3}$	(D)
			NA18924	31.27X	$1.32\;\times \;10^{-3}$	(D)
			NA19096	31.35X	$1.32\;\times \;10^{-3}$	(D)
		Toscani, Italy (TSI)	NA20754	31.63X	$1.04\;\times \;10^{-3}$	(D)
			NA20759	32.65X	$1.05\;\times \;10^{-3}$	(D)
			NA20766	29.95X	$1.03\;\times \;10^{-3}$	(D)
